# Crystal structure, DFT and Hirshfeld surface analysis of 2-amino-4-(2-chloro­phen­yl)-7-hy­droxy-4*H*-benzo[1,2-*b*]pyran-3-carbo­nitrile

**DOI:** 10.1107/S2056989019013537

**Published:** 2019-10-22

**Authors:** M. Beemarao, S. Silambarasan, A. Jamal Abdul Nasser, M. Purushothaman, K. Ravichandran

**Affiliations:** a Department of Physics, Kandaswami Kandar’s College, Velur, Namakkal 638 182, India; b Department of Chemistry, Jamal Mohamed College, Tiruchirappalli 620 020, India

**Keywords:** crystal structure, pyran, hydrogen bonding, Hirshfeld surface analysis, density functional theory

## Abstract

The benzo­pyran ring of the title com­pound, C_16_H_11_ClN_2_O_2_, is planar [maximum deviation = 0.079 (2) Å] and is almost perpendicular to the chloro­phenyl ring [dihedral angle = 86.85 (6)°]. In the crystal, N—H⋯O, O—H⋯N, C—H⋯O and C—H⋯Cl hydrogen bonds form inter- and intra­molecular inter­actions. The DFT/B3LYP/6-311G(d,p) method was used to determine the HOMO–LUMO energy levels. The mol­ecular electrostatic potential surfaces were investigated by Hirshfeld surface analysis and two-dimensional fingerprint plots were used to analyse the inter­molecular inter­actions in the mol­ecule.

## Abstract 2

The crystal and mol­ecular structures of the pyran derivative 2-amino-4-(2-chloro­phen­yl)-7-hy­droxy-4*H*-benzo[1,2-*b*]pyran-3-carbo­nitrile is reported. Hirshfeld surface analysis was performed on the mol­ecule and frontier orbitals were investigated with density functional theory calculations.

## Chemical context   

Pyran is an oxygen-containing heterocyclic group that exhibits various pharmacological activities. The pyran ring is a core unit in benzo­pyrans, chromones, flavanoids and coumarins. Numerous naturally-occurring com­pounds containing pyrans and benzo­pyrans show fascinating therapeutic activities, which include their use as anti­microbial (Khafagy *et al.*, 2002 ▸), anti­viral (Smith *et al.*, 1998 ▸; Martínez-Grau & Marco, 1997 ▸), mutagenicity (Hiramoto *et al.*, 1997 ▸), anti­proliferative (Dell & Smith, 1993 ▸), anti­tumour (Mohr *et al.*, 1975 ▸), anti­tuberculosis (Ferreira *et al.*, 2010 ▸), anti-HIV (He *et al.*, 2011 ▸), anti­fungal (Schiller *et al.*, 2010 ▸), anti­diabetic (Bisht *et al.*, 2011 ▸) and anti-inflammatory agents (Wang *et al.*, 1996 ▸, 2005 ▸). They are also used in cancer chemotherapy (Anderson *et al.*, 2005 ▸), in sex pheromone therapy (Bianchi & Tava, 1987 ▸) to control central nervous system activities (Eiden & Denk, 1991 ▸) and as calcium-channel antagonists (Shahrisa *et al.*, 2011 ▸),
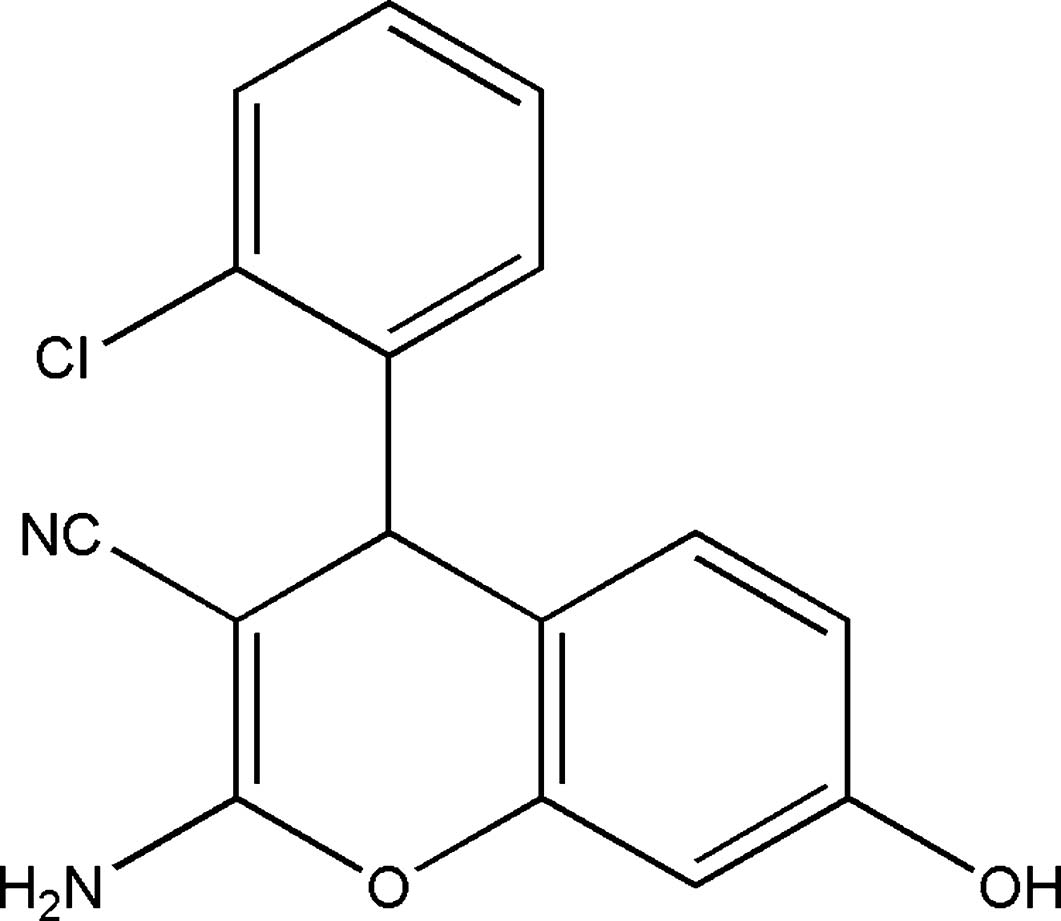


These attributes have prompted considerable research work in the synthetic field and inter­est in their structures, reactivities and biological properties. Against this background and to ascertain the structure of the title com­pound, namely 2-amino-4-(2-chloro­phen­yl)-7-hy­droxy-4*H*-benzo[1,2-*b*]pyran-3-carbo­nitrile, crystallographic studies have been carried out and are here reported.

## Structural commentary   

**Figure 1:**
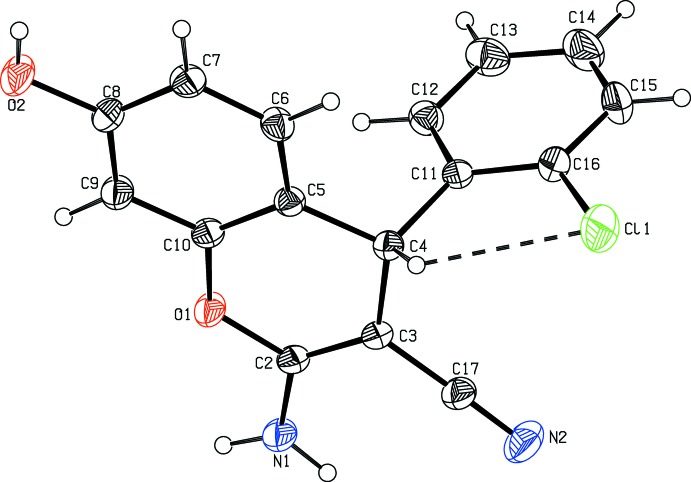
The mol­ecular structure of the title com­pound, showing the atom-numbering scheme and displacement ellipsoids drawn at the 30% probability level. The intra­molecular C4—H4⋯Cl1 hydrogen bond is drawn as a dashed line.

Fig. 1 ▸ shows the mol­ecular structure of the title mol­ecule and the intra­molecular C4—H4⋯Cl1 hydrogen bond. The chloro­phenyl-substituted benzo­pyran com­pound crystallizes in the monoclinic space group *P*2_1_/*c*. The benzo­pyran and chloro­phenyl rings in the mol­ecule are planar, as confirmed by the puckering parameters (Cremer & Pople, 1975 ▸) and asymmetry parameters *Q* = 0.101 (2) Å, θ = 105.6 (11)° and φ = 349.9 (14)° (Nardelli, 1983 ▸).

The bond lengths and angles are well within the expected limits and com­parable with literature values (Allen *et al.*, 1998 ▸). The plane of the benzo­pyran ring forms a dihedral angle of 86.85 (6)° with that of the chloro­phenyl ring and confirms the fact that the two moieties are in an axial orientation. The chloro­phenyl group is also planar, with a maximum deviation for atom C12 of −0.040 (1) Å. The orientation of the benzo­pyran and chloro­phenyl rings is also confirmed by the torsion angles C3—C4—C11—C12 = 76.5 (2)° and C3—C4—C11—C16 = −100.4 (2)°.

In the benzo­pyran system, the attached carbo­nitrile, amino and hy­droxy groups lie in the same plane, with a maximum deviation for atom N2 of −0.053 (2) Å. The sum of the bond angles around atom N1 of the pyran ring is in accordance with the *sp* ^2^-hybridization state (360°; Beddoes *et al.*, 1986 ▸).

## Supra­molecular features   

**Figure 2:**
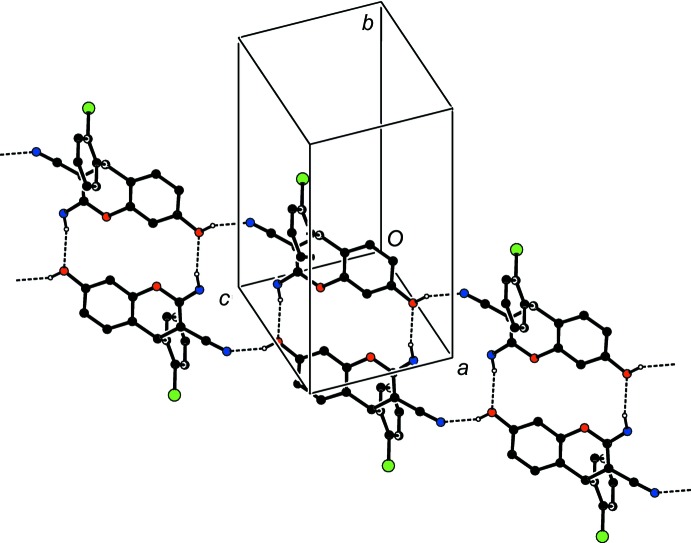
The crystal packing of the title com­pound, showing an 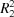
(16) motif and the *C*(8) chain formed *via* a pair of O—H⋯N and N—H⋯O hydrogen bonds.

**Figure 3:**
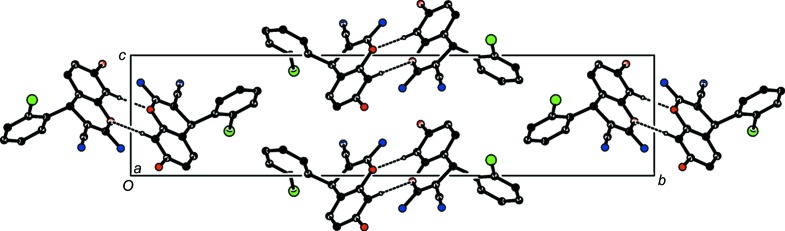
Part of the crystal structure showing the 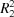
(8) dimers. H atoms not involved in hydrogen bonding (dashed lines) have been omitted for clarity.

**Figure 4:**
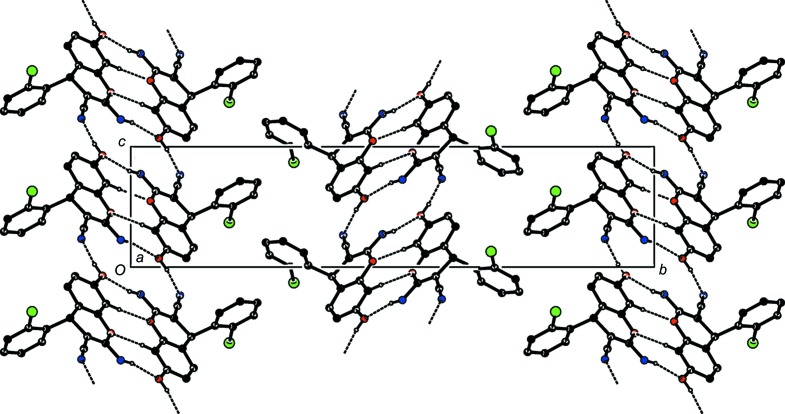
The overall crystal packing of the title com­pound, viewed along the *a*-axis direction.

The packing of the mol­ecules in the unit cell is stabilized by strong inter­molecular C—H⋯O, O—H⋯N and N—H⋯O hydrogen bonds (Table 1 ▸). The O2—H2⋯N2^ii^ inter­action leads to the formation of a *C*(10) chain running along the *a* axis. The mol­ecules are also linked by pairs of inter­molecular N1—H1*A*⋯O2^i^ and O2—H2⋯N2^ii^ hydrogen bonds, forming inversion dimers with 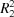
(16) ring motifs (Fig. 2 ▸) (Bernstein *et al.*, 1995 ▸), and the dimers are further connected by C9—H9⋯O1^i^ hydrogen bonds, forming 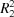
(8) rings along the *b*-axis direction, as shown in Fig. 3 ▸. Three C—H⋯π (Table 1 ▸) inter­actions com­plete the packing, forming a three-dimensional (3D) supra­molecular structure. The overall crystal packing of the title com­pound is shown in Fig. 4 ▸.

## Density functional theory (DFT) study   

**Figure 5:**
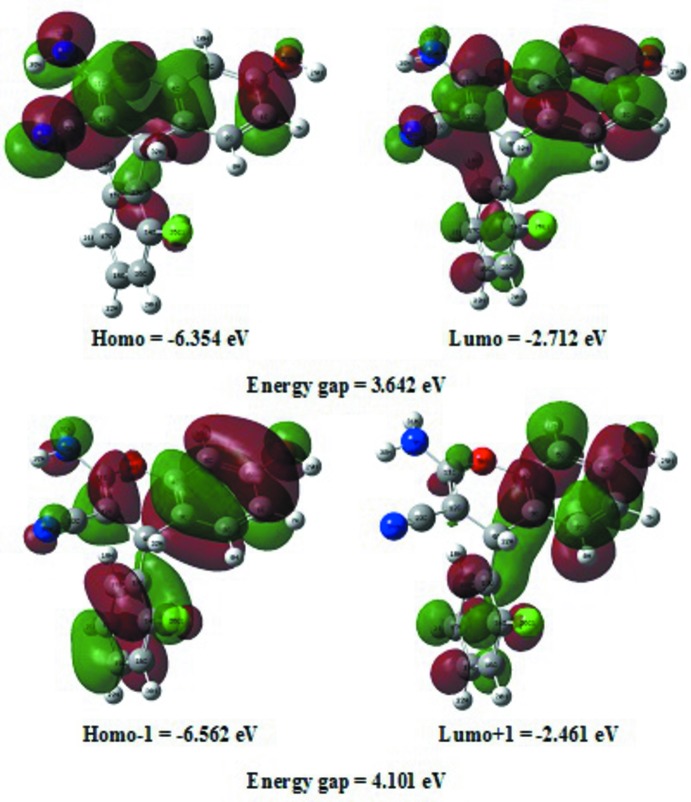
The frontier mol­ecular orbitals (FMOs) of the title com­pound.

The optimized mol­ecular structure and frontier mol­ecular orbitals (FMOs) were calculated using the DFT/B3LYP/6-311G(d,p) basis set implemented in the *GAUSSIAN09* program package (Frisch *et al.*, 2009 ▸). The highest occupied mol­ecular orbital (HOMO) and the lowest unoccupied mol­ecular orbital (LUMO) are called FMOs as they lie at the outermost boundaries of the electrons of the mol­ecules. The frontier orbital gap helps to characterize the chemical reactivity and the kinetic stability of the mol­ecule. A mol­ecule with a small frontier orbital gap is generally associated with a high chemical reactivity and a low kinetic stability, and is also termed a soft mol­ecule. The electron distribution of the HOMO-1, HOMO, LUMO and LUMO+1 energy levels and the energy values are shown in Fig. 5 ▸. The positive and negative phases are represented in green and red, respectively.

The HOMO of the title mol­ecule is localized on the entire mol­ecule except for the chloro­benzene ring, while the LUMO is located on the whole mol­ecule. However, the HOMO-1 is localized on the entire mol­ecule, with the LUMO+1 confined to the chloro­benzene and benzo­pyran rings, except for the amino substituent. The DFT study shows that the FMO energies, *i.e. E*_HOMO_ and *E*_LUMO_, are −6.354 and −2.712 eV, respectively, and the HOMO–LUMO energy gap is 3.642 eV. The title com­pound has a small frontier orbital gap, hence the mol­ecule has high chemical reactivity and low kinetic stability.

## Hirshfeld surface analysis   

**Figure 6:**
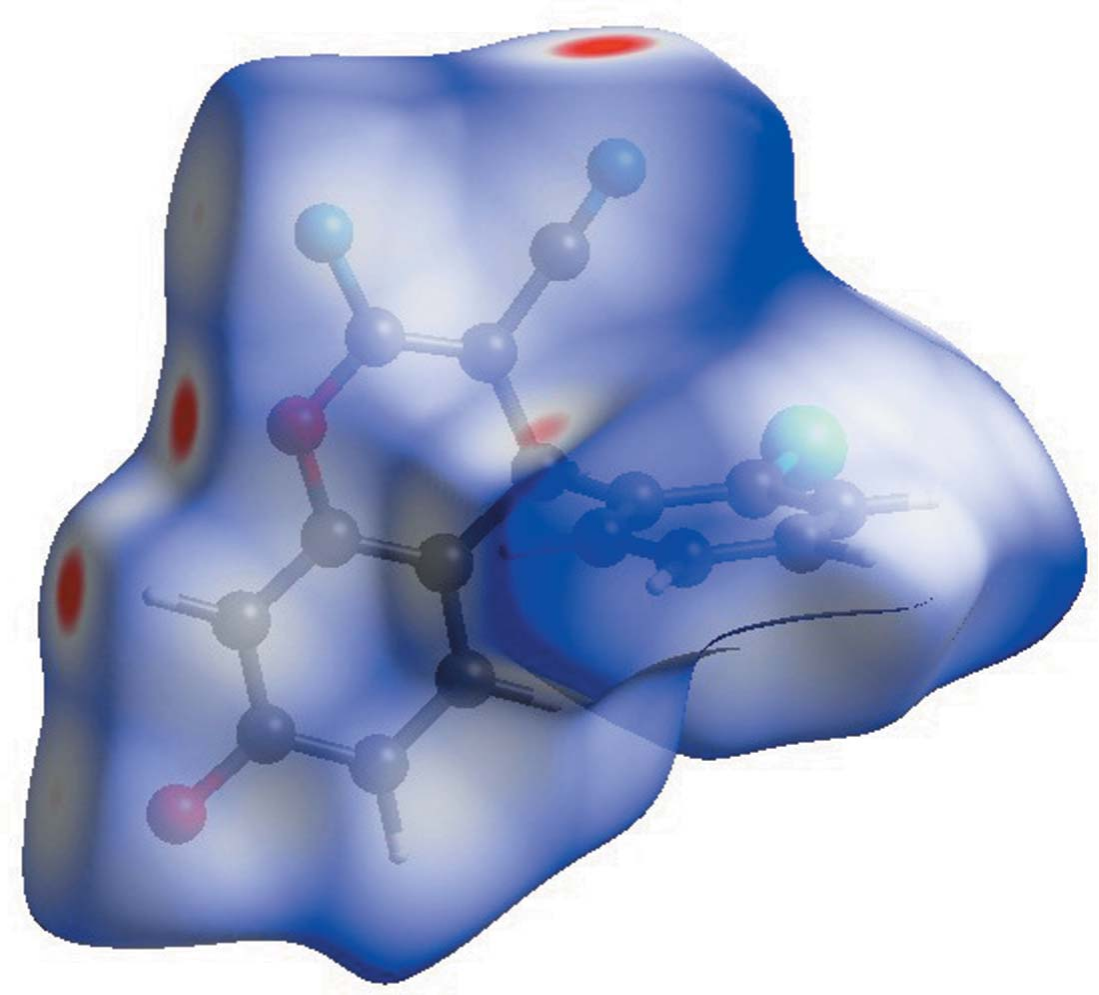
Hirshfeld surface mapped over *d*_norm_ in the range −0.6146 to 1.6047 a.u.

Hirshfeld surface analysis (Spackman & Jayatilaka, 2009 ▸) and two-dimensional (2D) fingerprint plots (McKinnon *et al.*, 2007 ▸) were performed and created with *CrystalExplorer17* (Turner *et al.*, 2017 ▸) for the idenfication of the inter­molecular inter­actions in the title com­pound. The Hirshfeld surface diagram mapped over *d*_norm_ is shown in Fig. 6 ▸. The 3D *d*_norm_ surfaces were plotted with a standard (high) surface resolution and are shown as blue and red regions around the atoms related with positive (hydrogen-bond donors) and negative (hydrogen-bond acceptors) electrostatic potentials, respectively.

**Figure 7:**
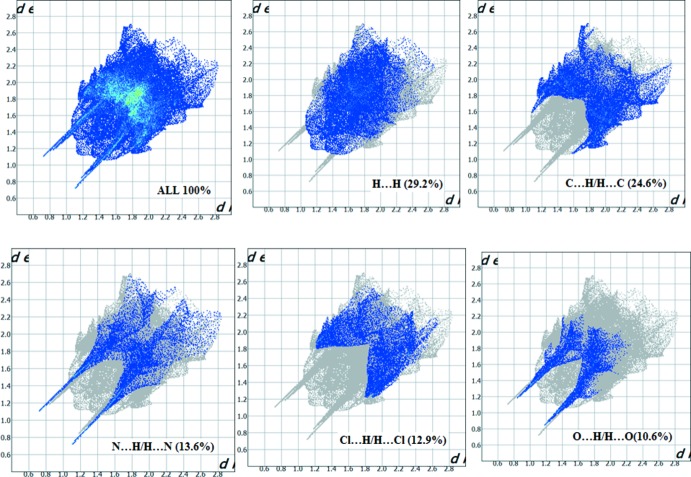
The 2D fingerprint plots for the title com­pound.

The 2D fingerprint plots of the *d*_i_ and *d*_e_ points for the contacts contributing to the Hirshfeld surface analysis are shown in Fig. 7 ▸. They indicate that inter­molecular H⋯H contacts provide the largest contribution (29.2%) to the Hirshfeld surface and the percentage contributions of the other inter­actions are C⋯H/H⋯C = 24.6%, N⋯H/H⋯N = 13.6%, Cl⋯H/H⋯Cl = 12.9% and O⋯H/H⋯O = 10.6%.

## Database survey   

A search of the Cambridge Structural Database (CSD, Version 5.40, update of November 2018; Groom *et al.*, 2016 ▸) for the 4*H*-benzo­pyran fragment revealed 10 hits where the fragment adopts a planar conformation. Nearly all the bond lengths in the title structure are the same within standard uncertainties as the corresponding values in the structure of 2-amino-4-(2-chloro­phen­yl)-7,7-dimethyl-5-oxo-5,6,7,8-tetra­hy­dro-4*H*-chromene-3-carbo­nitrile hemihydrate (CSD refcode LAPZIN; Hu *et al.*, 2012 ▸).

## Synthesis and crystallization   

A mixture of 2-chloro­benzaldehyde (6.2 g, 0.05 mol), malono­nitrile (3.3 ml, 0.05 mol) and resorcinol (5.5 g, 0.05 mol) in water (150 ml) was added to a 10% aqueous K_2_CO_3_ solution (10 ml) in a 250 ml round-bottomed flask. The resulting solution was refluxed for about 2 h. The progress of the reaction was monitored by thin-layer chromatography using silica gel-G plates. After product formation, the reaction mixture was kept in a refrigerator overnight. The solid mass that settled was filtered off by suction and washed well with a mixture of methanol and water, and finally dried in air. The resulting crude solid was recrystalized from methanol giving a white solid. The purified sample was recrystallized from 1,4-dioxane using the slow-evaporation method (m.p. 250–255 °C).

## Refinement   

Crystal data, data collection and structure refinement details are summarized in Table 2 ▸. H atoms were positioned geometrically (N—H = 0.88–0.90 Å and C—H = 0.93–0.98 Å) and allowed to ride on their parent atoms, with *U*_iso_(H) = 1.5*U*_eq_(C) for methyl H atoms and 1.2*U*_eq_(C) otherwise.

## Supplementary Material

Crystal structure: contains datablock(s) global, I. DOI: 10.1107/S2056989019013537/sj5577sup1.cif

Structure factors: contains datablock(s) I. DOI: 10.1107/S2056989019013537/sj5577Isup2.hkl

CCDC references: 1873687, 1873687

Additional supporting information:  crystallographic information; 3D view; checkCIF report

## Figures and Tables

**Table 1:** Hydrogen-bond geometry (Å, °) *Cg*1, *Cg*3 and *Cg*4 are the centroids of the O1/C2–C5/C10 ring, the C11–C16 ring and the benzo­pyran system, respectively.

*D*—H⋯*A*	*D*—H	H⋯*A*	*D*⋯*A*	*D*—H⋯*A*
N1—H1*A*⋯O2^i^	0.86	2.19	3.037 (2)	167
C9—H9⋯O1^i^	0.93	2.50	3.416 (2)	168
O2—H2⋯N2^ii^	0.82	1.99	2.773 (2)	160
C4—H4⋯Cl1	0.98	2.58	3.102 (2)	113
C12—H12⋯*Cg*1	0.93	2.74	3.085 (2)	103
C12—H12⋯*Cg*4	0.93	2.83	3.291 (2)	112
C14—H14⋯*Cg*3^iii^	0.93	2.85	3.494 (2)	127

Symmetry codes: (i) 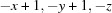; (ii) 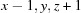; (iii) 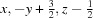.

**Table 2:** Experimental details

Crystal data	
Chemical formula	C_16_H_11_ClN_2_O_2_
*M*_r_	298.72
Crystal system, space group	Monoclinic, *P*2_1_/*c*
Temperature (K)	296
*a*, *b*, *c* (Å)	6.6658 (3), 30.1600 (16), 7.2193 (4)
β (°)	106.088 (2)
*V* (Å^3^)	1394.53 (12)
*Z*	4
Radiation type	Mo *K*α
μ (mm^−1^)	0.28
Crystal size (mm)	0.15 × 0.10 × 0.10

Data collection	
Diffractometer	Bruker SMART APEXII CCD
Absorption correction	Multi-scan (*SADABS*; Bruker, 2008 ▸)
*T*_min_, *T*_max_	0.959, 0.973
No. of measured, independent and observed [*I* > 2σ(*I*)] reflections	21888, 2941, 2115
*R*_int_	0.034
(sin θ/λ)_max_ (Å^−1^)	0.632

Refinement	
*R*[*F* ^2^ > 2σ(*F* ^2^)], *wR*(*F* ^2^), *S*	0.037, 0.103, 1.05
No. of reflections	2941
No. of parameters	191
H-atom treatment	H-atom parameters constrained
Δρ_max_, Δρ_min_ (e Å^−3^)	0.19, −0.29

Computer programs: *APEX2* (Bruker, 2008 ▸), *SAINT* (Bruker, 2008 ▸), *SHELXS97* (Sheldrick, 2008 ▸), *SHELXL2018* (Sheldrick, 2015 ▸), *ORTEP-3* (Farrugia, 2012 ▸), *SHELXL97* (Sheldrick, 2008 ▸) and *PLATON* (Spek, 2009 ▸).

## supplementary crystallographic information

### Crystal data

**Table:**

C_16_H_11_ClN_2_O_2_	*F*(000) = 616
*M**_r_* = 298.72	*D*_x_ = 1.423 Mg m^−^^3^
Monoclinic, *P*2_1_/*c*	Mo *K*α radiation, λ = 0.71073 Å
*a* = 6.6658 (3) Å	Cell parameters from 2115 reflections
*b* = 30.1600 (16) Å	θ = 2.7–26.7°
*c* = 7.2193 (4) Å	µ = 0.28 mm^−^^1^
β = 106.088 (2)°	*T* = 296 K
*V* = 1394.53 (12) Å^3^	Block, white crystalline
*Z* = 4	0.15 × 0.10 × 0.10 mm

### Data collection

**Table:**

Bruker SMART APEXII CCD diffractometer	2115 reflections with *I* > 2σ(*I*)
Radiation source: fine-focus sealed tube	*R*_int_ = 0.034
ω and φ scans	θ_max_ = 26.7°, θ_min_ = 2.7°
Absorption correction: multi-scan (*SADABS*; Bruker, 2008)	*h* = −6→8
*T*_min_ = 0.959, *T*_max_ = 0.973	*k* = −38→38
21888 measured reflections	*l* = −9→8
2941 independent reflections	

### Refinement

**Table:**

Refinement on *F*^2^	Hydrogen site location: inferred from neighbouring sites
Least-squares matrix: full	H-atom parameters constrained
*R*[*F*^2^ > 2σ(*F*^2^)] = 0.037	*w* = 1/[σ^2^(*F*_o_^2^) + (0.0392*P*)^2^ + 0.5426*P*] where *P* = (*F*_o_^2^ + 2*F*_c_^2^)/3
*wR*(*F*^2^) = 0.103	(Δ/σ)_max_ = 0.001
*S* = 1.05	Δρ_max_ = 0.19 e Å^−^^3^
2941 reflections	Δρ_min_ = −0.28 e Å^−^^3^
191 parameters	Extinction correction: SHELXL2018 (Sheldrick, 2015), Fc^*^=kFc[1+0.001xFc^2^λ^3^/sin(2θ)]^-1/4^
0 restraints	Extinction coefficient: 0.0061 (13)

### Special details

**Table:**

Geometry. All esds (except the esd in the dihedral angle between two l.s. planes) are estimated using the full covariance matrix. The cell esds are taken into account individually in the estimation of esds in distances, angles and torsion angles; correlations between esds in cell parameters are only used when they are defined by crystal symmetry. An approximate (isotropic) treatment of cell esds is used for estimating esds involving l.s. planes.
Refinement. Refinement of F^2^ against ALL reflections. The weighted R-factor wR and goodness of fit S are based on F^2^, conventional R-factors R are based on F, with F set to zero for negative F^2^. The threshold expression of F^2^ > 2sigma(F^2^) is used only for calculating R-factors(gt) etc. and is not relevant to the choice of reflections for refinement. R-factors based on F^2^ are statistically about twice as large as those based on F, and R- factors based on ALL data will be even larger.

### Fractional atomic coordinates and isotropic or equivalent isotropic displacement parameters (Å^2^)

**Table:**

	*x*	*y*	*z*	*U*_iso_*/*U*_eq_	
C2	0.9057 (3)	0.54802 (6)	−0.1320 (2)	0.0342 (4)	
C3	1.0377 (3)	0.58264 (5)	−0.0758 (2)	0.0334 (4)	
C4	1.0200 (3)	0.61567 (5)	0.0772 (2)	0.0314 (4)	
H4	1.156330	0.618094	0.172872	0.038*	
C5	0.8634 (3)	0.59885 (5)	0.1766 (2)	0.0314 (4)	
C6	0.8329 (3)	0.62022 (6)	0.3376 (3)	0.0379 (4)	
H6	0.915142	0.644648	0.387878	0.045*	
C7	0.6839 (3)	0.60615 (6)	0.4248 (3)	0.0408 (4)	
H7	0.665264	0.621197	0.531266	0.049*	
C8	0.5627 (3)	0.56954 (6)	0.3525 (3)	0.0379 (4)	
C9	0.5916 (3)	0.54711 (6)	0.1960 (3)	0.0398 (4)	
H9	0.512626	0.522076	0.148322	0.048*	
C10	0.7398 (3)	0.56246 (6)	0.1111 (2)	0.0335 (4)	
C11	0.9601 (3)	0.66108 (5)	−0.0142 (2)	0.0322 (4)	
C12	0.7556 (3)	0.66946 (6)	−0.1204 (3)	0.0400 (4)	
H12	0.654979	0.647668	−0.127966	0.048*	
C13	0.6976 (4)	0.70909 (7)	−0.2149 (3)	0.0539 (5)	
H13	0.559727	0.713738	−0.285481	0.065*	
C14	0.8442 (4)	0.74176 (7)	−0.2045 (4)	0.0621 (6)	
H14	0.805563	0.768466	−0.268905	0.075*	
C15	1.0465 (4)	0.73504 (7)	−0.0995 (4)	0.0600 (6)	
H15	1.145714	0.757164	−0.091365	0.072*	
C16	1.1027 (3)	0.69495 (6)	−0.0052 (3)	0.0446 (5)	
C17	1.1869 (3)	0.58941 (6)	−0.1782 (3)	0.0402 (4)	
O1	0.7539 (2)	0.53801 (4)	−0.04856 (18)	0.0420 (3)	
O2	0.4103 (2)	0.55394 (5)	0.4287 (2)	0.0552 (4)	
H2	0.406537	0.569128	0.521925	0.083*	
Cl1	1.36070 (9)	0.68831 (2)	0.12960 (12)	0.0757 (2)	
N1	0.9057 (3)	0.51897 (5)	−0.2734 (2)	0.0455 (4)	
H1A	0.816166	0.497735	−0.298713	0.055*	
H1B	0.995343	0.521634	−0.338516	0.055*	
N2	1.3050 (3)	0.59408 (7)	−0.2655 (3)	0.0608 (5)	

### Atomic displacement parameters (Å^2^)

**Table:**

	*U*^11^	*U*^22^	*U*^33^	*U*^12^	*U*^13^	*U*^23^
C2	0.0376 (10)	0.0333 (9)	0.0359 (9)	0.0032 (7)	0.0174 (8)	−0.0023 (7)
C3	0.0336 (9)	0.0322 (9)	0.0385 (10)	0.0028 (7)	0.0169 (8)	−0.0012 (7)
C4	0.0314 (9)	0.0305 (9)	0.0334 (9)	−0.0007 (7)	0.0110 (7)	−0.0026 (7)
C5	0.0339 (9)	0.0306 (9)	0.0310 (9)	0.0025 (7)	0.0113 (7)	0.0014 (7)
C6	0.0474 (11)	0.0339 (9)	0.0335 (9)	−0.0043 (8)	0.0132 (8)	−0.0054 (7)
C7	0.0556 (12)	0.0392 (10)	0.0332 (10)	0.0003 (9)	0.0215 (9)	−0.0050 (8)
C8	0.0423 (10)	0.0419 (10)	0.0349 (10)	−0.0008 (8)	0.0198 (8)	0.0007 (8)
C9	0.0442 (11)	0.0394 (10)	0.0403 (10)	−0.0071 (8)	0.0192 (9)	−0.0085 (8)
C10	0.0391 (10)	0.0340 (9)	0.0308 (9)	0.0008 (7)	0.0155 (8)	−0.0042 (7)
C11	0.0384 (10)	0.0298 (8)	0.0329 (9)	−0.0008 (7)	0.0172 (8)	−0.0043 (7)
C12	0.0434 (11)	0.0373 (10)	0.0395 (10)	−0.0014 (8)	0.0117 (9)	−0.0021 (8)
C13	0.0610 (14)	0.0490 (12)	0.0493 (12)	0.0128 (10)	0.0113 (10)	0.0079 (10)
C14	0.0862 (18)	0.0437 (12)	0.0623 (15)	0.0089 (12)	0.0303 (13)	0.0165 (11)
C15	0.0759 (16)	0.0375 (11)	0.0783 (16)	−0.0104 (11)	0.0407 (14)	0.0059 (11)
C16	0.0445 (11)	0.0405 (10)	0.0554 (12)	−0.0057 (8)	0.0248 (10)	−0.0037 (9)
C17	0.0408 (11)	0.0408 (10)	0.0433 (11)	−0.0024 (8)	0.0188 (9)	−0.0082 (8)
O1	0.0484 (8)	0.0419 (7)	0.0444 (7)	−0.0136 (6)	0.0274 (6)	−0.0159 (6)
O2	0.0659 (10)	0.0596 (9)	0.0547 (9)	−0.0182 (7)	0.0409 (8)	−0.0158 (7)
Cl1	0.0392 (3)	0.0653 (4)	0.1207 (6)	−0.0156 (3)	0.0190 (3)	−0.0037 (4)
N1	0.0532 (10)	0.0422 (9)	0.0511 (10)	−0.0077 (8)	0.0309 (8)	−0.0147 (7)
N2	0.0571 (12)	0.0748 (13)	0.0627 (12)	−0.0146 (10)	0.0368 (10)	−0.0200 (10)

### Geometric parameters (Å, º)

**Table:**

C2—N1	1.345 (2)	C9—H9	0.9300
C2—O1	1.347 (2)	C10—O1	1.3932 (19)
C2—C3	1.353 (2)	C11—C16	1.385 (2)
C3—C17	1.408 (2)	C11—C12	1.389 (2)
C3—C4	1.516 (2)	C12—C13	1.377 (3)
C4—C5	1.509 (2)	C12—H12	0.9300
C4—C11	1.525 (2)	C13—C14	1.375 (3)
C4—H4	0.9800	C13—H13	0.9300
C5—C10	1.375 (2)	C14—C15	1.367 (3)
C5—C6	1.393 (2)	C14—H14	0.9300
C6—C7	1.382 (3)	C15—C16	1.387 (3)
C6—H6	0.9300	C15—H15	0.9300
C7—C8	1.382 (3)	C16—Cl1	1.737 (2)
C7—H7	0.9300	C17—N2	1.146 (2)
C8—O2	1.366 (2)	O2—H2	0.8200
C8—C9	1.376 (2)	N1—H1A	0.8600
C9—C10	1.379 (2)	N1—H1B	0.8600
			
N1—C2—O1	110.58 (15)	C5—C10—C9	123.36 (16)
N1—C2—C3	126.39 (16)	C5—C10—O1	122.36 (15)
O1—C2—C3	123.03 (15)	C9—C10—O1	114.28 (15)
C2—C3—C17	116.70 (15)	C16—C11—C12	116.58 (17)
C2—C3—C4	123.38 (15)	C16—C11—C4	123.24 (16)
C17—C3—C4	119.72 (15)	C12—C11—C4	120.11 (15)
C5—C4—C3	109.23 (13)	C13—C12—C11	121.94 (18)
C5—C4—C11	112.01 (13)	C13—C12—H12	119.0
C3—C4—C11	109.81 (13)	C11—C12—H12	119.0
C5—C4—H4	108.6	C14—C13—C12	119.8 (2)
C3—C4—H4	108.6	C14—C13—H13	120.1
C11—C4—H4	108.6	C12—C13—H13	120.1
C10—C5—C6	116.37 (15)	C15—C14—C13	120.1 (2)
C10—C5—C4	122.16 (15)	C15—C14—H14	120.0
C6—C5—C4	121.44 (15)	C13—C14—H14	120.0
C7—C6—C5	121.86 (17)	C14—C15—C16	119.5 (2)
C7—C6—H6	119.1	C14—C15—H15	120.3
C5—C6—H6	119.1	C16—C15—H15	120.3
C6—C7—C8	119.46 (16)	C11—C16—C15	122.1 (2)
C6—C7—H7	120.3	C11—C16—Cl1	120.11 (15)
C8—C7—H7	120.3	C15—C16—Cl1	117.79 (16)
O2—C8—C9	116.76 (16)	N2—C17—C3	178.0 (2)
O2—C8—C7	123.03 (16)	C2—O1—C10	118.87 (13)
C9—C8—C7	120.21 (16)	C8—O2—H2	109.5
C8—C9—C10	118.71 (17)	C2—N1—H1A	120.0
C8—C9—H9	120.6	C2—N1—H1B	120.0
C10—C9—H9	120.6	H1A—N1—H1B	120.0
			
N1—C2—C3—C17	1.4 (3)	C4—C5—C10—O1	1.5 (3)
O1—C2—C3—C17	−179.18 (17)	C8—C9—C10—C5	1.0 (3)
N1—C2—C3—C4	176.25 (17)	C8—C9—C10—O1	−178.75 (16)
O1—C2—C3—C4	−4.3 (3)	C5—C4—C11—C16	138.04 (17)
C2—C3—C4—C5	10.3 (2)	C3—C4—C11—C16	−100.42 (19)
C17—C3—C4—C5	−175.06 (16)	C5—C4—C11—C12	−45.1 (2)
C2—C3—C4—C11	−112.94 (19)	C3—C4—C11—C12	76.49 (19)
C17—C3—C4—C11	61.7 (2)	C16—C11—C12—C13	1.1 (3)
C3—C4—C5—C10	−8.7 (2)	C4—C11—C12—C13	−176.05 (17)
C11—C4—C5—C10	113.14 (18)	C11—C12—C13—C14	−0.3 (3)
C3—C4—C5—C6	172.89 (16)	C12—C13—C14—C15	−0.5 (3)
C11—C4—C5—C6	−65.2 (2)	C13—C14—C15—C16	0.5 (3)
C10—C5—C6—C7	−1.2 (3)	C12—C11—C16—C15	−1.0 (3)
C4—C5—C6—C7	177.30 (17)	C4—C11—C16—C15	175.98 (18)
C5—C6—C7—C8	0.8 (3)	C12—C11—C16—Cl1	178.20 (13)
C6—C7—C8—O2	−179.04 (18)	C4—C11—C16—Cl1	−4.8 (2)
C6—C7—C8—C9	0.5 (3)	C14—C15—C16—C11	0.3 (3)
O2—C8—C9—C10	178.20 (17)	C14—C15—C16—Cl1	−178.97 (18)
C7—C8—C9—C10	−1.4 (3)	N1—C2—O1—C10	175.17 (15)
C6—C5—C10—C9	0.3 (3)	C3—C2—O1—C10	−4.3 (3)
C4—C5—C10—C9	−178.19 (17)	C5—C10—O1—C2	5.7 (3)
C6—C5—C10—O1	179.99 (16)	C9—C10—O1—C2	−174.53 (16)

### Hydrogen-bond geometry (Å, º)

*Cg*1, *Cg*3 and *Cg*4 are the centroids of the O1/C2–C5/C10 and C11–C16 rings, and the benzopyran system, respectively.

**Table:**

*D*—H···*A*	*D*—H	H···*A*	*D*···*A*	*D*—H···*A*
N1—H1*A*···O2^i^	0.86	2.19	3.037 (2)	167
C9—H9···O1^i^	0.93	2.50	3.416 (2)	168
O2—H2···N2^ii^	0.82	1.99	2.773 (2)	160
C4—H4···Cl1	0.98	2.58	3.102 (2)	113
C12—H12···*Cg*1	0.93	2.74	3.085 (2)	103
C12—H12···*Cg*4	0.93	2.83	3.291 (2)	112
C14—H14···*Cg*3^iii^	0.93	2.85	3.494 (2)	127

Symmetry codes: (i) −*x*+1, −*y*+1, −*z*; (ii) *x*−1, *y*, *z*+1; (iii) *x*, −*y*−1/2, *z*−1/2.
